# Editorial: Mitochondrial and lysosomal dysfunction in neurodegenerative diseases: Molecular mechanisms and therapeutic strategies

**DOI:** 10.3389/fnagi.2022.1029440

**Published:** 2022-10-04

**Authors:** Chen Xiang, Xiao Yousheng, Zhu Xiaolei, Yang Wenjie, Pu Jiali, Pei Zhong

**Affiliations:** ^1^Department of Neurology, The First Affiliated Hospital of Guangzhou Medical University, Guangzhou, China; ^2^Department of Neurology, The First Affiliated Hospital of Guangxi Medical University, Nanning, China; ^3^Department of Neurology, Affiliated Drum Tower Hospital of Nanjing University Medical School, Nanjing, China; ^4^Department of Neurology, University of Maryland, Rockville, MD, United States; ^5^Department of Neurology, Second Affiliated Hospital of Zhejiang University, Hangzhou, China; ^6^Department of Diagnostic Radiology and Nuclear Medicine, The First Affiliated Hospital of Sun Yet-sen University, Guangzhou, China

**Keywords:** mitochondrial dysfunction, lysosomal dysfunction, neurodegenerative disease, molecular mechanism, therapeutic

Neurodegenerative diseases have a serious effect on the daily lives of patients and generate health-related direct and indirect societal economic costs (Bourdenx et al., 2017). Alzheimer's disease (AD), Parkinson's disease (PD), and amyotrophic lateral sclerosis (ALS) are prevalent age-related neurodegenerative diseases, characterized by severe neuronal loss and deposition of misfolding proteins in disease-specific brain regions (Yan et al., 2013). Currently, the precise mechanism of neurodegenerative diseases is unclear. However, more and more research has begun to indicate lysosomes and mitochondria as playing significant roles in neurodegenerative diseases, meaning they may be promising therapeutic targets in future.

In the clinic, brain metabolism of glucose and oxygen were found at a low level in AD patients detected by PET-CT, which were associated with mitochondria dysfunction. In addition, several studies have indicated that mitochondrial morphology and mitochondrial DNA were altered, and respiratory chain function was impaired in AD patients and animal models. Extracellular Aβ and intracellular tau protein accumulation have also been proposed to be bi-directionally linked to mitochondrial dysfunction (Monzio et al., 2020).

Mitochondrial abnormalities have also found to be a common occurrence in both sporadic and familial PD and presented as mitochondrial electron transport chain impairment, alterations in mitochondrial morphology and dynamics, mitochondrial DNA mutations, and calcium homeostasis anomalies. In addition, *SCNA, PRKN, PINK1*, and *PARK7* were reported as susceptibility genes in PD and they enhance PD risk by leading to mitochondrial dysfunction (Subramaniam and Chesselet, 2013).

As for lysosomes, impairment of the autophagy-lysosomal pathway (ALP) has been regarded as a hallmark of AD and PD. The ALP plays a significant role in cellular protein degradation, including pathological protein α-synuclein and amyloid beta, and thus maintains the homeostasis of neurons (Martini-Stoica et al., 2016). The importance of ALP impairment in PD is further supported by genetic studies. It is reported that *GBA, LRRK2, SNCA, ATP13A2, VPS35*, and *FBXO7* were closely related to PD (Zhang et al., 2018).

This Research Topic discusses molecular mechanisms of mitochondrial and lysosomal dysfunction in AD and PD, and further explores therapeutic strategies targeting mitochondrial and lysosomal function for prevention and precise treatment. We here present five articles covering a broad span of original research and scientific reviews within these Research Topics.

Liang et al. summarized the role of oxidative stress, mitochondrial biogenesis, dynamics, and mitophagy involved in the aging process and AD, and the result of dysfunctional mitochondria and corresponding exercise interventions. Furthermore, they concluded the underlying mechanisms of corresponding interventions that affect these processes.

Zhao et al. explored the association between rare variants of 69 lysosomal storage disorder (LSD) genes and PD in 3,879 patients and 2,931 controls from Parkinson's Disease and Movement Disorders Multicenter Database and Collaborative Network in China (PD-MDCNC) using next-generation sequencing, confirming the importance of lysosomal mechanisms in PD pathogenesis.

Li H. et al. reported three novel mutations (c.1103A > G/p.D368G, c.1696C > G/p.L566V, and c.1470delC/p.R490fs494X) in *PANK2* and indicated a correlation between phenotype and mitochondrial dysfunction, providing new insight for evaluating the clinical severity of patients based on the degree of mitochondrial dysfunction.

Zheng and Fan discussed *GBA* mutations that increase the risk of developing PD and focused on the role of glucocerebrosidase deficiency in PD pathogenesis. Finally, they discussed the implications for PD therapy based on the relationship between *GBA* and PD.

Li P. et al. found that growth/differentiation factor-15 (GDF15) protected rotenone-treated SH-SY5Y cells from toxicity by preserving mitochondrial function and decreasing apoptosis by upregulating PGC1α *via* p53. And these anti-apoptotic effects may be mediated by the PI3K/Akt/mTOR signaling pathway.

Gong et al. found Wen-Shen-Jian-Pi (WSJP) prescription could delay the decline in motor function of ALS model mice by reducing the degeneration of neurons and may be a potential treatment for ALS in the clinic.

Taken together, the articles presented in this Research Topic of Frontiers in Aging Neuroscience examine several susceptibility genes related to lysosome and mitochondria in AD, PD, and ALS. Furthermore, these basic research studies of AD, PD, and ALS could offer novel insights into what could become viable treatments for neurodegenerative diseases.

## Author contributions

All authors listed have made a substantial, direct, and intellectual contribution to the work and approved it for publication.

## Conflict of interest

The authors declare that the research was conducted in the absence of any commercial or financial relationships that could be construed as a potential conflict of interest.

## Publisher's note

All claims expressed in this article are solely those of the authors and do not necessarily represent those of their affiliated organizations, or those of the publisher, the editors and the reviewers. Any product that may be evaluated in this article, or claim that may be made by its manufacturer, is not guaranteed or endorsed by the publisher.
